# Luteinizing hormone changes in gonadotropin-releasing hormone antagonist protocol in in vitro fertilization cycles: A cross-sectional study

**DOI:** 10.18502/ijrm.v17i3.4520

**Published:** 2019-05-29

**Authors:** Batool Hosein Rashidi, Roya Kabodmehri, Mamak Shariat, Ensieh Shahrokh Tehraninejad, Alireza Abdollahi, Maryam Bagheri, Fedieh Hagholahi

**Affiliations:** Health Reproductive Research Center, Imam Khomeini Hospital, Tehran University of Medical Sciences, Tehran, Iran.

**Keywords:** *Gonadotropin-releasing hormone-antagonist*, * Luteinizing hormone*, * In vitro fertilization.*

## Abstract

**Background:**

Because of the unexpected and often dramatic inhibition of luteinizing hormone (LH) secretion related with the usage of gonadotropin-releasing hormone (GnRH)-antagonist, there has been a probable need for exogenous LH supplementation. There is a basic and clinical evidences that show late development of follicle needs an LH but there is a threshold for LH requirements during folliculogenesis.

**Objective:**

The purpose of this study was to evaluate the changes in serum LH and the identification of patients who benefit from the addition of LH.

**Materials and Methods:**

Seventy volunteers for antagonist protocol in IVF cycle were enrolled in this prospective cross-sectional study. The study was carried out in Reproductive Health Research Center, University of Medical Sciences between July 2016 and February 2016. Serum LH level was estimated 24 h before and after the first (GnRH) antagonist injection. The primary outcome was the serum level of LH and its change in the three groups and the secondary outcome was Egg and Embryo quality.

**Results:**

LH changes above or below 50% had no effect on the number of follicle, the number of oocyte, Germinal vesicle oocyte, metaphase 1 oocyte, metaphase 2 oocyte, endometrial thickness, and chemical and clinical pregnancy.

**Conclusion:**

We evaluated the changes of serum LH in the patients who were entered in the antagonist protocol. Our study showed no significant difference in LH levels 24 h before and after the injection of the antagonist between the three groups, and LH changes did not affect the outcome of pregnancy.

## 1. Introduction

According to the two-cell-gonadotropin hypothesis, androgen plays a crucial role in making of estradiol (E2) by granulosa cell. Luteinizing hormone (LH) directly contributes to the control of granulosa cell function (1). The role of LH in folliculogenesis includes stimulation of theca cell, androgen production, triggering ovulation, supporting the corpus luteum, and development of late follicular stage (2). There is a basic and clinical evidence that show late development of follicle needs an LH, but there is a threshold for LH requirements during folliculogenesis (1). It is known that < 1% of LH receptor needs to be occupied to provoke a best steroidogenic response (3), thus resting level of LH (1–10 IU/L) must be adequate to offer maximal stimulation to theca cell (3).

The role of in vitro fertilization was accepted as an effective way for the treatment of infertile patients (4). It has been well-known that suppressing the release of a pituitary follicle stimulatory hormone (FSH) and LH in controlled ovarian hyper stimulation cycles with Gonadotropin-releasing hormone (GnRH) analogs is an important factor relevant to the success of IVF treatment. In some IVF cycle, premature surge was a big problem. This problem was solved by Schally in 1971 through introduction of a GnRH agonist (5). Although GnRH-agonist managed premature LH surge, it was associated with creating new problems such as occurrence of cyst formation at the start of induction ovulation and ovarian hyper stimulation syndrome (OHSS) (1). The use of GnRH agonist associated with long duration of administration, low E2 level and side effect due to estrogen deficiency (1). Despite the availability of GnRH antagonist, it was not used for this purpose due to allergies in 1971 (6). In the antagonist protocol, several important advantages may be noted which include the following: in the polycystic ovarian syndrome (PCOS) patients, GnRH agonist can be used as a trigger in this protocol and reduce the risk of OHSS (7, 8). Several studies compared this two analog and the use of GnRH-antagonist was associated with shorter stimulation period and decreased the occurrence of OHSS by more than 50% (9, 10). One of the problems with taking GnRH antagonist seems to be a reduction in the blood level of LH.

There is a wide range of pituitary responses to GnRH antagonists. In some patients that received a single dose of GnRH-antagonist (3 mg), LH level may be decreased below a threshold level (9, 11). Some studies showed that the fast response to all doses of GnRH antagonists cause a drop in LH levels, which is similar in among all doses. However, a large change in LH levels is detected for the pituitary recovery 24 hr later. While, 24 hr after injection the low antagonist doses allow a quick recovery and high doses result are a partial recovery in pre-treatment LH levels (12). Maximum patients have a normal response; however, “hypo responders” might ovulate prematurely, while “over-suppressed” patients may have impaired follicular development (12).

Therefore, the aim of this study is the evaluation of LH change in antagonist protocol and identify patients who benefit from adding LH.

## 2. Materials and Methods

In this prospective cross-sectional study, 70 volunteers for antagonist protocol in IVF cycle (due to PCOS, a poor responder in the previous cycle and Egg donor) who were referred to the Reproductive Health Research Center, Tehran University of Medical Sciences between July 2016 and February 2016 were enrolled. PCOS defined by Rotterdam criteria: clinical and/or biochemical hyperandrogenism and oligo-amenorrhea and/or ultrasonographic criteria ≥ 12 follicles (2–9 mm diameter) and/or ovarian volume ≥ 10 mL (1). Based on ESHRE meeting the poor responder patients are defined according to two criteria of the following criteria:

• Advanced maternal age (≥ 40 y) or any other risk factor.
• A previous POR (cycles canceled or ≤ 3 oocytes with a conventional protocol).
• An abnormal ovarian reserve test (antral follicle count < 5–7 follicles or anti-Mullerian hormone (AMH) < 0.5–1.1 ng/ml) (13).

Exclusion criteria was allergy to antagonist and patients with long agonist protocol. On the first day of menstrual cycle, suppression of ovary was confirmed by ultrasonography. The hormonal assessment was performed on the 2nd day of menstrual cycle including FSH, LH, and E2. For all patients gonadotropin (Follistim, Merck; Gonal-F, EMD-Serono) was started from the 2nd day of menstruation according to AMH and antral follicular countage, previous response to gonadotropin. Six days after the start of gonadotropin, ultrasonography was performed for estimate follicular size and repeated if necessary. When follicle reached ≥ 13 mm, we used GnRH-antagonist (Cetrotide, EMD-Serono 0.25 mg/day SC). Serum LH level was estimated 24 hr before and after first Cetrotide injection. When two follicles were < 18 mm, we used *Human chorionic gonadotropin* (10.000 IU; Pregnyl, Organon, Oss, Netherlands) intramuscular as a trigger; 36 hr after trigger, oocyte retrieval was performed under ultrasound guide. Primary outcome was the serum level of LH and its change in the three groups and the secondary outcome was Egg and Embryo quality.

### Ethical consideration

The study was approved by the Ethics Committee Review Board of Tehran University (No: IRTUMS.VCR.REC.1395.1405). Written inform consent was obtained from all participants.

### Statistical analysis

The data were analyzed using Statistical Package for the Social Sciences, version 20, SPSS Inc, Chicago, Illinois, USA (SPSS) software. Independent t-test, Man–Whitney test, and Kruskal–Wallis was used to compare the parametric and nonparametric parameters between groups. Comparison of qualitative variables between groups was performed using Chi-square test. The quantitative parameters were reported as mean ± SEM. A p < 0.05 was taken as statistically significant. Spearman correlation test was used to assess the correlation of LH parameters with other factors.

## 3. Results

**Figure 1:**
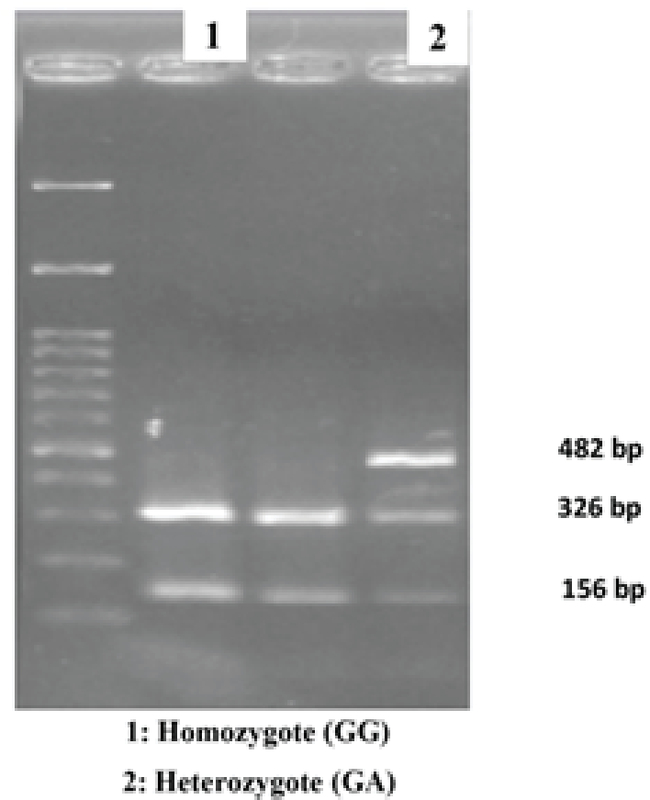
Slope or shot in the PCO is significantly higher than the other two groups, except in accordance with the above figure are more tangible between PCO and Normal.

Table I shows the demographic characteristics and basal Hormonal assay. As expected, the average age of the poor responder group is more than two other groups (37.8 ± 4.43) (p = 0.001). According to Kruskal–Wallis (nonparametric test), the serum level of basal LH that was measured on the second day of menstruation shows significant difference (p = 0.004). As expected serum level of LH is higher in PCOS (6.7 ± 4.3) than poor responder (5.18 ± 3) and normal responder groups (3.67 ± 4) (Figure 1). As well as the level of AMH is high in PCOS groups. Other variables examined (basal FSH, E2, BMI, duration of infertility, number of previous IVF) in three groups showed no significant difference. Results of Table II show the significant differences between the three groups containing follicle-number, oocyte number, metaphase 2 (M2) oocyte, Germinal vesicle (GV) oocyte, number of transferred embryos and the number of consumed Gonadotropin ampule. To determine the difference between the three groups, we used Post Hoc and Bonferroni test. The number of follicles, GV oocyte and transferred embryo show significant difference between the PCO and poor responder group and the PCO and normal responder only, but there was no difference between Poor and normal responder. The number of the eggs and M2 oocyte has only significant difference between the PCO and Poor responder patients. This difference cannot be seen in each other. But it was significantly different between the three groups together in the number of gonadotropins ampule, and gonadotropin consumed in poor responder group was more than other groups (p = 0.0001). There was no significant difference (p = 0.45, p = 0.843) in LH levels 24 h before and after the injection of the antagonist between the three groups. As well as, the LH ratio and LH change not differ in the three groups (p = 0.077, p = 0.486). According to the Chi-square test, the LH difference percent in the three groups was not significant (p = 0.81). Spearman correlation test was used to assess the correlation of LH parameters with the number of follicle and oocyte and GV oocyte, metaphase 1 (M1) oocyte, M2 oocyte, endometrial thickness. The only significant relationship was seen between LH ratio and M1 (r = 0.31, p = 0.008) and LH change and M1(r = 0.26, p = 0.025). In other words, if the LH (24 hours after administration of the antagonist) is high, that is, the LH suppression by the antagonist is lower and the rate of M1 oocyte was more. According to Mann–Whitney and Fisher test, the correlation of the LH decrease were not significantly associated with GV, M1, M2 oocyte, the number of the follicle, the number of the oocyte, endometrial thickness (Table III). In the other words, the lower levels of LH after the injection had no effect on outcomes.

**Table:** Characteristics of patients

**Treatment Group**	**Group I n = 20**	**Group II n = 23**	**Group III n = 27**	**p-value**
**Variables**		
Age (y)	37.8 ± 4.43	30.73 ± 4.82	32.55 ± 5.84	0.001
BMI (kg/m${}^{2}$)	27.23 ± 3.02	25.65 ± 3.65	25.63 ± 2.25	0.43
infertility Duration (y)	6.35 ± 4	6.08 ± 5.04	4.67 ± 4.56	0.46
FSH (Iu/ml)	7.4 ± 3.1	6.56 ± 6.45	6.07 ± 2.80	0.08
LH base (Iu/ml)	5.18 ± 3	6.7 ± 4.3	3.67 ± 1.35	0.004
E2	41.15 ± 16.4	41.73 ± 7.2	41.7 ± 15.2	0.08
AMH	0.98 ± 0.97	8.7 ± 5.16	2.8 ± 1.4	0.001
TSH	3.11 ± 2.48	2.45 ± 1.14	2.29 ± 1.16	0.60
ViT D	22.11 ± 13.13	18.16 ± 14.09	19.75 ± 12.52	0.29
Number of previous IVF (N, %)	0.21 ± 0.53	0.17 ± 0.38	0.18 ± 0.39	0.96
Note: Data presented as mean ± SD; these parameters were analyzed with Kruskal-Wallis (nonparametric)				

BMI: Body mass index	FSH: Follicle stimulating hormone	LH: Luteinized Hormone
E2: Estradiol	AMH: Anti Mullerian Hormone	TSH: Thyroid Stimulating Hormone
ViT D: Vitamin D	IVF: in vitro fertilization	Group1: Poor responder
Group2: PCOS	Group3: Normal responder	

**Table:** Treatment outcomes in a patient in three groups

**Treatment Group**		**Group I n = 20**	**Group II n = 23**	**Group III n = 27**	**p-value**
**Variables**			
LH (24 hr before antagonist)*		3.2 ± 3.89	4.1 ± 4.8	3.11 ± 1.60	0.45
LH(24 hr after antagonist)*		2.68 ± 1.83	2.55 ± 1.10	2.64 ± 1.42	0.843
LH ratio*		1.67 ± 2.3	0.89 ± 0.42	0.89 ± 0.44	0.077
LH change*		–0.55 ± 3.6	–1.5 ± 4.8	–0.47 ± 1.14	0.486
LH decrease*		0.67 ± 2.3	–0.11 ± 0.42	–0.10 ± 0.44	0.077
No. follicles *		4.45 ± 3.2	17.43 ± 7.62	7.66 ± 3.9	0.001
No. Oocyte*		2.8 ± 2.58	12.08 ± 5.96	5.11 ± 3.29	0.001
Quality oocyte*			
	M1	0.35 ± 0.58	0.69 ± 1.1	0.29 ± 0.66	0.494
	M2	0.35 ± 0.58	9.7 ± 6.2	4.44 ± 2.96	0.001
	GV	0.2 ± 0.41	1.06 ± 2.16	0.37 ± 1.0	0.004
Endometrial thickness (mm)*		7.5 ± 1.7	7.5 ± 1.3	7.64 ± 1.5	0.839
Days of Stimulation*		10.15 ± 2.8	10.8 ± 4.3	9.7 ± 2.4	0.578
Number of Gonadotropin*		4 ± 0	2.26 ± 0.61	3.25 ± 0.85	0.0001
Cycle cancelation **			0.153
	Yes	7 (35%)	10 (43.5%)	5 (18.5%)	
	NO	13 (65%)	13 (56.5%)	22 (81.5%)	
Total number of embryo*		1.4	8 ± 5.4	2.37 ± 2.3	0.001
Embryo transfer**			0.099
	Yes	8 (42)	4 (17.5)	12 (44.5)	
	No	11 (58)	19 (82.5)	15 (55.5)	
Embryo quality*			
	Lower than 8 cell	6 (66.7)	22 (95.7)	16 (80)	0.09
	8 cell	4 (44)	15 (65.2)	13 (65)	0.51
	Morolla	0	0	3 (5.8)	0.07
	Blastocyst	0	1 (4.3)	0	0.68
LH decrease**			
	> 50%	5 (26)	3 (13)	1 (4)	0.081
	< 50%	14 (74)	20 (87)	26 (96)	
Note: *data presented as mean ± SD and these parameters were analyzed with Kruskal–Wallis (nonparametric **data presented as n(%), and these parameters were analyzed with Fisher's exact test or Chi-square Test					

LH: Luteinized Hormone	M1: Metaphase 1	M2: Metaphase 2
GV: Germinal vesicle	LH change: LH after antagonist- LH before the antagonist	
LH ratio: LH after antagonist/LH before antagonist	Group1: Poor responder	
Group2: PCOS	Group3: normal responder	

**Table 3:** Correlation of LH decreases percent with treatment outcome in total group


**Treatment Group**	**> 50% (n = 14)**	**< 50% (n = 23)**	**p-value**
**Variables**		
M1	0.21 ± 0.42	0.50 ± 0.94	0.39
M2	4.07 ± 4.35	5.24 ± 5	0.11
GV	1.2 ± 2.1	0.61 ± 1.32	0.30
Oocyte number	5.4 ± 5.8	7.2 ± 5.6	0.13
Endometrial thickness (mm)	7.6 ± 1.9	7.6 ± 1.2	0.99
Total number of the embryo	2.8 ± 3.6	4.1 ± 4.7	0.28
Note: Data presented as mean ± SD. Data analyzed with Mann-Whitney test (nonparametric)			
M1: Metaphase 1 M2: Metaphase 2 GV: Germinal vesicle			

## 4. Discussion

We evaluate the changes of serum LH in the three groups of patients who were entered in antagonist protocol. In none of the patients in the three groups, the blood level of the LH followed the GnRH antagonist reached below one. There was no reduction in more than 50% of the LH levels in any group following a GnRH antagonist. In our study, there was no significant difference in the LH levels 24 h before and after injection of the antagonist between three groups, as well as on the LH ratio we didn't find a significant difference. Changes in LH more than 50% (drop more than 50% or even increase) was not significantly associated with GV, M1, M2 oocyte, the number of the follicle, the number of the oocyte, endometrial thickness. Several studies have evaluated the role of LH in antagonist protocol (14). Studies in hypogonadotropic patients using r-FSH established that FSH can induce follicular development in the preovulatory phase, but E2 and androstenedione remain very low, and this advocates that follicular development depends on the action of LH to induce androgen biosynthesis as a substrate for aromatase activity (15). Suitable folliculogenesis and steroid genesis are necessary for successful fertilization and implantation. Therefore, it depends upon a certain threshold level of LH. Although, the amount of LH necessary for standard follicle and oocyte maturation is not known (16), it is likely to be very low since a best steroidogenic response can be prompted when < 1% of follicular LH receptors are occupied (16). There is also evidence that excessive level of LH can have an adverse effect on follicular development (17). So, there is a window for LH while the low and high level of LH is a harmful effect in follicular maturation (18). This is an update of a Cochrane review in 2001, and previously updated in 2006 and 2011 (19). In the study by Huirne and coworkers, they evaluated the change of plasma level in LH in five groups with different dosage of GnRH–antagonist, and effect of these changes on IVF outcome. In this study, no pregnancies were observed in relation to either very high or low LH which protects the window hypothesis (20). According to this study, excessive fluctuation in LH levels might disrupt this balance and associate with adverse effect (20). Overall it seems that LH may have a valuable effect through a mechanism that recovers oocyte maturation (20). In the studies by Gomez–Palomares and coworkers (1) and Lisi and coworker, significant improvement in IVF outcome in poor responder patients was found (21). Because of the unexpected and often dramatic inhibition of LH secretion related with the usage of GnRH-anta, there has been a probable need for exogenous LH supplementation (1). The meta analysis from six clinical randomized trials showed that normogomadotropic patients did not benefit from adding LH (22). In the cohort retrospective study in poor responder or AMA (advanced maternal age) did not protect add LH supplementation (22). In the study by Hill and coworkers, adding exogenous LH in AMA patients improved implantation and pregnancy rate (23). In the study by Kol, patients who were hyper sensitive to GnRH–antagonist benefitted from r-LH (12). In the study by Younis and coworkers, r-LH supplementation to r-FSH following GnRH-antagonist administration did not seem to significantly increase serum E${}_{2}$ level on the day of Human chorionic gonadotropin administration in AMA patients. This recommends that endogenous serum LH levels following GnRH-antagonist beginning are adequate for suitable late follicular ovarian steroidogenesis (14). The novelty of our study is evaluating the LH change in three subgroups including hyper responder (PCOS patients), hypo responds (poor ovarian reserve), and normal responder (donor patients).

##  Limitation

Limitations of the study:

• Small sample size may affect results; however, further clinical trial studies with large sample size are needed.
• Because in some patients we had frozen embryos, there was no possibility of assessing pregnancy.

## 5. Conclusion

The results showed no difference in the levels of LH and changes of LH in antagonist protocol between three groups, so it is not practical to assess the LH level in ART cycles.

##  Conflict of Interest 

In this study there was no conflict of interest.
